# Testicular Inflammatory Myofibroblastic Tumor: A Known Entity at a Very Rare Site

**DOI:** 10.1155/2017/1410843

**Published:** 2017-12-21

**Authors:** Hans-Ullrich Voelker, Daniel Kuehn, Annette Strehl, Stefan Kircher

**Affiliations:** ^1^Institute of Pathology, Leopoldina Hospital, 97422 Schweinfurt, Germany; ^2^Department of Urology, Leopoldina Hospital, 97422 Schweinfurt, Germany; ^3^Institute of Pathology, University of Wuerzburg, 97080 Wuerzburg, Germany

## Abstract

Inflammatory myofibroblastic tumors (IMT) are distinctive lesions of unknown etiology, composed of myofibroblastic spindle cells with an associated inflammatory background. They can occur in a wide age range and at all anatomic sites, but most frequently they can be observed in the lung (especially in pediatric cases), abdomen, or retroperitoneum. The urinary bladder is one of the most common sites in urological cases. We present a very rare case of IMT of the testis. Clinically, a 40-year-old patient showed a palpable painless lesion of the right testis. Ultrasound examination showed two solid intratesticular foci. During surgical intervention, the intraoperative frozen section revealed mesenchymal tumors admixed with an uncommon inflammatory infiltrate, consistent with a reorganized abscess. Despite the benign result, orchiectomy was performed due to the multifocal presentation and the large size of 3 cm. The final diagnosis was IMT without ALK-rearrangement. Incomplete resection increases the risk of local relapses to 30%. In this case, a complete resection could be achieved and the patient is free of tumor 15 months later.

## 1. Introduction

Inflammatory myofibroblastic tumors (IMT), formerly known as plasma cell granuloma or inflammatory pseudotumor, are rare benign tumors which can occur at all anatomic sites. The most frequent location in pediatric cases is the lung, whereas in adults IMT are often located in the abdomen or retroperitoneum. Manifestations at uncommon sites are also possible, for example, in the larynx [1, 2]. In urological cases, IMT mostly affect the urinary bladder and are sometimes associated with a history of preceding iatrogenic manipulation [3]. Despite the wide possible range of patient age and anatomic distribution, IMT is a rare entity. The etiology remains unclear. Around 50% of IMT show a clonal rearrangement of the ALK gene (2p23, anaplastic lymphoma kinase). ALK-negative tumors are more often found in elderly patients and show a higher degree of cytological pleomorphism and mitotic activity as well as a higher risk for local relapses, especially after incomplete surgical resection [4]. Because some patients can benefit from treatment with both steroidal and nonsteroidal anti-inflammatory medication, an aberrant inflammatory response was assumed to be important for pathogenesis, in combination with a true neoplastic nature of the disease. Therapy of choice is a complete excision.

Microscopic examination shows a mesenchymal tumor dominated by well demarcated fascicular proliferations of myofibroblastic spindle cells admixed with plasma cells, lymphocytes, and histiocytes. Additional immunohistochemical investigations can be helpful for the correct diagnosis due to a wide spectrum of morphological variants. These tumors present a pitfall during intraoperative frozen section due to the wide spectrum of possible mimics ranging from simple reactive postinflammatory changes to true spindle cell tumors.

## 2. Case Presentation

**Figure 1:**
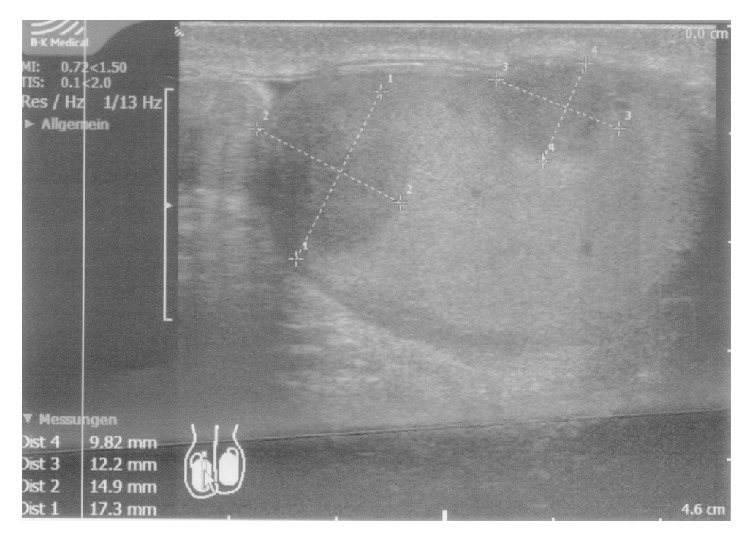
Ultrasound of testis. Two tumors were detectable.

A 40-year-old patient (nonsmoker, no other complaints) reported painless palpable lesions of the left and right testis. Ultrasound revealed small inconspicuous cystic lesions which were not suspicious of a neoplastic process. Serum AFP (2.5 (<7.0) ng/ml) and beta-HCG (<0.1 (<2.6) IU/l) were not elevated. Three months later, clinical controls resulted in a distinctly palpable tumor of the right testis, but no changes in left testis. Ultrasound examination yielded two tumors within the right testis with hypoechoic signals, suspicious of a malignant testicular tumor (Figure 1). Therefore, surgical intervention was indicated.

During orchiectomy, intraoperative frozen sections were performed. The first microscopic analysis revealed reactive cystic changes of the left epididymis with associated fibrosis and mild chronic inflammation. The lesion of the right testis showed an uncommon but nonspecific microscopic pattern, so that a final intraoperative diagnosis was not possible. The histological features were not typical of any common type of malignant or nonmalignant testicular tumor, but consistent with a previous local inflammation or organized abscess. Because of the large size of the lesion, orchiectomy was performed.

**Figure 2:**
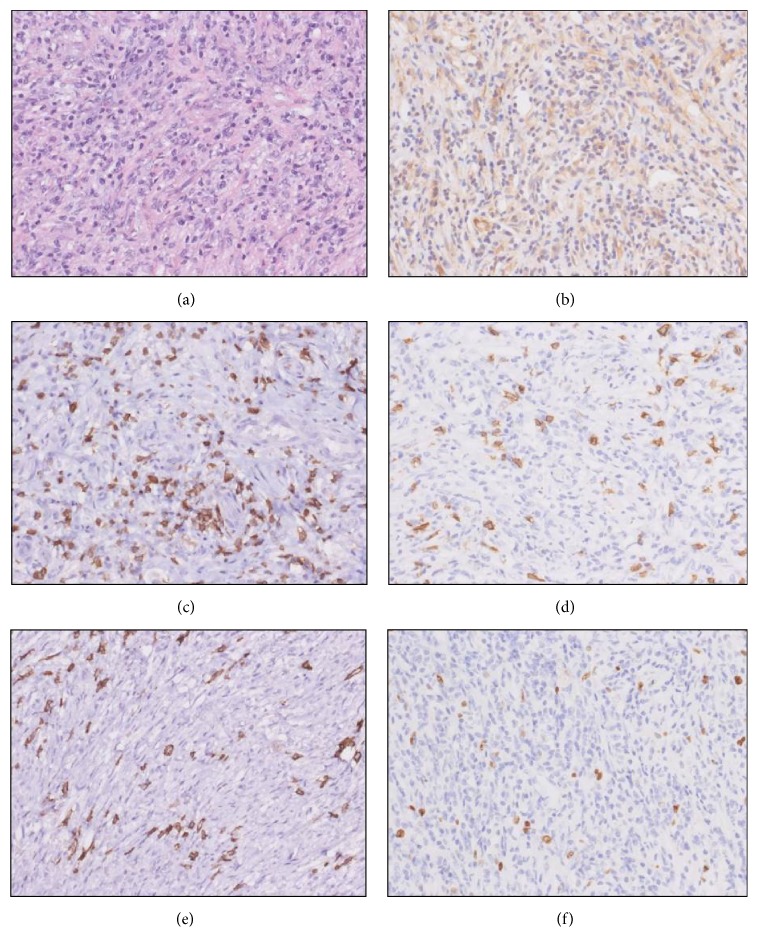
Histology and immunohistochemistry: (a) HE ×200, (b) positivity for sm-actin, ×200, (c) few T-cells in CD5, ×200, (d) scattered B-cells in CD20, ×200, (e) also scattered plasma cells in CD138, negative for IgG4 (not shown), ×200, and (f) very low proliferation, Ki67, ×200.

On gross examination, the testis showed two bright-colored intratesticular tumors with the largest diameter of 3 cm. The surrounding parenchyma was largely regular. Histology and immunohistochemistry on paraffin-embedded tissue revealed a mesenchymal tumor surrounded by mild chronic inflammatory changes. The HE-stain displayed a well demarcated proliferation of myofibroblastic spindle cells with irregular storiform arrangement and loosely admixed collagenous fibers. Bland inconspicuous nuclei could be observed with only very few mitoses. The spindle cell population was accompanied by focally dense infiltrates of lymphocytes and plasma cells as well as scattered ganglion-like cells. Adjacent seminiferous tubules were severely atrophic with so-called Sertoli-only syndrome. The epididymis showed moderate chronic inflammatory changes with cystic dilation of ducts and no evidence of spermatogenesis; no pathologic features could be found in the surrounding soft tissue and the spermatic cord. Immunohistochemical staining for smooth muscle actin was diffusely positive within the tumor; S100 was only expressed in dendritic cells. No specific reaction was found with desmin, CD34, Pankeratin MNF116, and EMA. Among the inflammatory cells, there were less B-lymphocytes (CD20 positive) than T-lymphocytes (CD5 positive). CD30 was expressed in isolated activated cells without atypical morphology; CD56 displayed loosely distributed ganglion-like cells. ALK-1 was not expressed. The plasma cell population (CD138+) did not show a significant overgrowth with IgG4+ cells. Proliferative activity (MiB-1) was not significantly increased. Additionally, for safety PLAP stains were done despite absence of suspicious changes within seminiferous tubules. Additional germ cell neoplasia in situ was excluded by negative stain for PLAP. Some examples are given in Figure 2.

All stains and technical processes were performed according to routine protocols (Autostainer Bond Max, Leica). Antibodies were tested using suitable positive controls according to the manufacturer's recommendation.

EBER (EBV-encoded RNA) in situ hybridization was performed (EBER probe Y-5200, PNA-ISH-Detection Kit K-5201, Dako) with a negative result.

FISH-analysis demonstrated a lack of ALK-gene (2p23.3) alterations in 97% of the cells using an ALK break-apart probe, corresponding to a negative result. In approximately 34% of cells only one colocalization signal could be observed. A negative result was also noted using a ROS1 break-apart probe where 98% of cells showed no evidence of gene-rearrangement in the ROS1-gene (6q22). In 17% of cells, a gain of up to 4 colocalization signals could be observed, whereas 27% displayed only one colocalization signal.

In conclusion, final diagnosis of inflammatory myofibroblastic tumor (IMT) of the right testis was established, with two separate foci 3 cm apart and associated moderate chronic inflammatory changes as well as fibrosis with secondary cystic change of the epididymis. The diagnosis was confirmed by a second opinion at the Institute of Pathology of the University of Wuerzburg (Director: Professor Dr. med. A. Rosenwald).

## 3. Discussion

Inflammatory myofibroblastic tumor (IMT) is a benign tumorous proliferation of myofibroblastic spindle cells accompanied by a mixed chronic inflammatory infiltrate consisting of plasma cells and lymphocytes. The clinical presentation with suspicion for malignancy is not uncommon and fast-growing processes are possible. In some cases large tumors are locally aggressive with destruction of adjacent structures [2]. The etiology of IMT remains unknown, a review of the relevant literature suggesting more than one possible pathway of pathogenesis: a more truly neoplastic pathway in ALK-positive cases and a nonneoplastic reactive pathway secondary to inflammation in ALK-negative cases.

A possible stimulation of proliferative activity as a result of autoimmune processes has also been discussed [5]. Despite the wide range of possible anatomic locations, most tumors were detected in the lung, the abdomen, or the retroperitoneum [4]. A few case reports describe lesions of the paratesticular soft tissue or spermatic cord [6–9]. Intratesticular manifestation is very rare, with only three reports existing [10–12]. In all these cases solitary tumors were observed, whereas we could verify two separate manifestations in the same testis. In our case report no medical history of relevant recurrent inflammatory diseases of the scrotum, epididymis, or testis existed; furthermore the patient did not specify previous trauma or other relevant diseases (e.g., hernia, undescended testis, and extreme sportive activity). Hematological investigations did not reveal any pathological findings, especially no signs of persistent inflammation with elevation of leucocytes or CRP. However, the microscopically evident moderate chronic unspecific inflammation and fibrosis of the epididymis could be seen as an indication of an underlying reactive pathogenesis.

Even though epididymitis with consecutive orchitis is a common occurrence in elderly patients, there are only very few reports of additional IMT at this site. In the majority of cases a purely reactive lesion seems unlikely. Molecular studies could detect genetic aberrations including a clonal origin, chromosomal abnormalities involving 2p23, fusion of the ALK gene with tropomyosin 3 (TPM3-ALK) or tropomyosin 4 (TPM4-ALK), translocations of ROS1 gene, RET mutations, or alterations using comparative genomic analysis CGH [2, 13–16]. In this setting, the morphologic subtypes defined by Coffin et al. (nodular fasciitis-like, fibrous histiocytoma-like, and collagen dense with a pattern similar to desmoid fibromatosis) could represent cases of true neoplastic nature rather than reactive lesions with “plasma cell granuloma” at one end of the spectrum. The latter could conversely be triggered by an atypical autoimmune response. The importance of Alk-1 expression for distinction between neoplastic and reactive etiology remains unclear. Anaplastic lymphoma kinase (Alk-1) expression is mostly detectable in IMFTs of patients under 40 years. Therefore, the hypothesis of a postinflammatory or traumatic origin could become important if the majority of Alk-1 negative cases were found to represent the nonneoplastic reactive variant. This could explain why Coffin et al. described a recurrence rate of 45% in Alk-positive and only 20% of Alk-negative pulmonary and extrapulmonary IMT [4]. Our case was negative for both ALK and ROS1.

Despite the often benign clinical course, local recurrence is possible, whereas the metastatic potential seems very low [4]. Cases with metastasis are rare and to our knowledge the relevant literature gives no evidence of true metastasis in contrast to multifocal development. After surgical excision the risk of recurrence is extremely small; therefore surgery is the therapy of choice with no additional aggressive treatment being necessary.

## 4. Conclusion

IMT is a rare entity, which needs special efforts of pathologist. The differential diagnosis of IMT comprises low grade myofibroblastic sarcomas as well as a long list of benign, reactive, or neoplastic spindle cell lesions, such as leiomyoma, solitary fibrous tumor, spindle cell carcinoma, nodular fasciitis, peripheral nerve sheath tumor, and sex cord stromal tumors. Morphology and immunohistochemical profile are helpful in ruling out these entities also at uncommon anatomic sites like testis. Complete excision is the therapy of choice.
